# Measles Vaccination Coverage After a Postelimination Outbreak

**DOI:** 10.1001/jamanetworkopen.2025.33732

**Published:** 2025-09-24

**Authors:** Rosemary A. Martoma, Joshua C. Martoma, Maimuna S. Majumder

**Affiliations:** 1 Department of Pediatrics, Harvard Medical School, Boston, Massachusetts; 2 Division of General Pediatrics, Boston Children’s Hospital, Boston; 3 KidsMates Inc, Boca Raton, Florida; 4 Florida Atlantic University, Schmidt College of Medicine, Boca Raton; 5 College of Computing, Data Science, and Society, University of California, Berkeley; 6 Department of Pediatrics, Harvard Medical School, Boston, Massachusetts; 7 Computational Health Informatics Program, Boston Children’s Hospital, Boston, Massachusetts

## Abstract

**Importance:**

Sustaining measles elimination in the US requires high population-level vaccination coverage. Postelimination outbreaks expose persistent immunity gaps, especially in underimmunized communities. Understanding vaccination trends after such outbreaks is critical for public health responses and preparedness.

**Objective:**

To evaluate changes in measles-mumps-rubella (MMR) vaccination coverage after a large postelimination measles outbreak in a major US pediatric primary care population.

**Design, Setting, and Participants:**

This repeated, surveillance cross-sectional study was conducted at an extensive central Ohio pediatric primary care network that served approximately 150 000 children at the following 3 time points: outbreak onset (October 8, 2022), 12 months (October 8, 2023), and 20 months (June 8, 2024). Children younger than 15 years with 1 well-child visit or more in the prior 24 months were analyzed. Children of Somali descent were analyzed as a prespecified subgroup.

**Exposure:**

Receipt of valid MMR vaccine doses per Centers for Disease Control and Prevention guidelines.

**Main Outcomes and Measures:**

Primary outcomes were (1) timely MMR vaccine 1 receipt by 16 months of age, (2) timely MMR vaccine 2 receipt 28 days or more after MMR vaccine 1 and by 84 months of age, and (3) receipt of 1 valid MMR dose or more by 84 months of age. Coverage was assessed at each time point. Risk differences were estimated using binomial generalized linear models with an identity link. The secondary outcome was MMR coverage stratified by Somali descent.

**Results:**

At 20 months after measles onset, the cohort included 149 092 children (median [IQR] age, 7.96 [4.23-11.50] years; 76 469 [51.3%] male; 12 880 [8.6%] Somali descent and 136 212 [91.4%] non-Somali descent). Timely MMR vaccine dose 1 coverage remained unchanged (66 143 of 123 490 [53.6%] at 0 months and 74 157 of 138 301 [53.6%] at 20 months; risk difference, 0.1 percentage points; 95% CI, –0.3 to 0.4 percentage points; *P* = .76). Timely MMR vaccine dose 2 coverage increased modestly (44 210 of 76 410 [57.9%] at 0 months and 50 875 of 84 576 [60.2%] at 20 months; risk difference, 2.3 percentage points; 95% CI, 1.7-2.8 percentage points; *P* < .001). Receipt of at least 1 MMR dose by 84 months of age increased from 59 049 of 76 410 (77.3%) at 0 months to 65 891 of 84 576 (77.9%) at 20 months (risk difference, 0.6 percentage points; 95% CI, 0.2-1.0 percentage points; *P* = .003). Children of Somali descent had significantly lower timely MMR vaccine dose 1 coverage at all time points (overall risk difference at baseline, –19.7 percentage points; 95% CI, –20.7 to –18.8 percentage points; *P* < .001).

**Conclusions and Relevance:**

In this repeated cross-sectional study of 149 092 children in a large central Ohio primary care network during the 20 months after outbreak onset, all measures of MMR coverage remained well below the 93% herd immunity threshold. These persistent, population-wide immunity gaps suggest the need for sustained, equity-focused public health strategies to maintain measles elimination.

## Key Points

**Question:**

Did measles vaccination coverage in an underimmunized central Ohio primary care network change at 12 and 20 months after a large postelimination outbreak?

**Findings:**

In this cross-sectional study of 149 092 children, timely measles-mumps-rubella (MMR) vaccine dose 1 coverage did not change; however, timely MMR vaccine dose 2 and overall coverage by 84 months of age increased slightly. Children of Somali descent had persistently lower timely MMR vaccine dose 1 coverage, with a widening gap.

**Meaning:**

Persistent, systemwide MMR immunity gaps in an extensive pediatric network suggest the need for targeted and sustained public health strategies to prevent future outbreaks and protect a quarter-century of US measles elimination.

## Teaser

This cross-sectional study evaluates whether measles-mumps-rubella vaccination coverage changed after a measles outbreak in children in central Ohio.

## Introduction

Measles is a highly contagious, vaccine-preventable disease that requires high population-level immunity to prevent sustained transmission.^1^ Among children older than 12 months, the measles-mumps-rubella (MMR) vaccine is estimated to be 93% effective after 1 dose and 97% effective after 2 doses.^2^ In the World Health Organization Region of the Americas, measles vaccination coverage must exceed 93% to maintain herd immunity.^3^ Although the US declared measles eliminated in 2000, the country continues to experience large (≥50 cases) postelimination outbreaks.^4^ These outbreaks disproportionately affect communities with persistent underimmunization and reflect vulnerabilities in national and local immunity.^3,4^

The Centers for Disease Control and Prevention (CDC) recommends routine MMR vaccination at 12 to 15 months for the first dose (MMR1) and at 4 to 6 years for the second dose (MMR2).^2^ With an accelerated schedule, the second dose may be administered 28 or more days after the first and count toward series completion.^2^ During outbreaks, infants aged 6 to 11 months may also receive an MMR dose; however, this dose does not count toward series completion.^2^

The CDC most commonly estimates MMR vaccination coverage using kindergarten-entry assessments, which combine administrative reporting and data from state immunization information systems.^5^ Schools submit aggregated vaccination and exemption data; however, these assessments exclude homeschooled children, nonreporting schools, and those younger than school-entry age.^5^ Immunization information systems are centralized electronic registries that compile vaccination records from clinical practitioners, but participation is voluntary in some states, including Ohio.^3^ Due to reporting gaps, delays, and a lack of individual-level verification, these systems are not well suited for real-time surveillance or outbreak response.

The 2022 to 2023 central Ohio measles outbreak resulted in 90 confirmed cases between June 12 and December 24, all in children younger than 15 years.^6^ Eighty-four of these cases were identified through an extensive Columbus-based pediatric primary care network (PCN), and early transmission was concentrated among children of Somali descent.^6^ Columbus Public Health (CPH) declared a local measles outbreak on November 9, 2022, after confirming multiple locally acquired cases.^6^ Although no definitive index case was confirmed, Martoma et al^3^ modeled an international importation on October 8, 2022, as the most plausible index case based on its epidemiologic linkage to the first locally acquired case.

In response to the outbreak, public health officials implemented a range of interventions, including outbreak notifications, quarantines, daycare closures, and walk-in vaccination clinics.^6^ However, widespread vaccine hesitancy limited MMR vaccine uptake.^7^ CPH estimated jurisdictional MMR vaccination coverage at 80% to 90% during the outbreak, although the methods used to generate this estimate were not described.^7^ In contrast, Martoma et al^3^ modeled 2-dose MMR coverage at outbreak onset as 53% (95% credible interval, 21%-77%) in the exposed population, which included children not yet age-eligible for MMR vaccination.

Given that 93% of outbreak cases were detected among children in this PCN,^6^ the analysis provides a detailed assessment of actual vaccination coverage in a high-risk, large-scale pediatric population. This study uses electronic medical records (EMRs) from an extensive PCN in central Ohio to assess MMR vaccination coverage over time, including timely receipt of MMR1 and MMR2, and identifies persistent postoutbreak immunity gaps. The primary objective was to evaluate whether coverage changed after the measles outbreak. A secondary objective was to compare coverage between children of Somali descent and their peers, given the early concentration of outbreak cases in this population.

## Methods

### Study Design, Setting, and Participants

This repeated cross-sectional surveillance study evaluated MMR vaccination coverage among children receiving care in a large central Ohio PCN. Coverage was assessed at 3 time points: outbreak onset (October 8, 2022), 12 months later (October 8, 2023), and 20 months later (June 8, 2024). The initial time point corresponds to the modeled date of the index case, preceding the official outbreak declaration.^3,6^ Children were included at each time point if they were younger than 15 years on the surveillance date and had at least 1 documented well-child visit in the preceding 24 months. The age cutoff was selected to align with the epidemiologic profile of the outbreak, in which all confirmed cases occurred among children younger than 15 years.^6^ The analytic cohort was reconstructed at each time point to account for changes in age and care engagement, reflecting a repeated cross-sectional design. Separate age-based criteria were applied to determine eligibility for each vaccination outcome. This study used fully deidentified EMR data and was reviewed by the Nationwide Children’s Hospital Institutional Review Board, which determined that it did not constitute human subjects research because it involved only secondary analysis of existing, deidentified data with no direct participant contact. All analyses were conducted in accordance with institutional privacy policies and the Strengthening the Reporting of Observational Studies in Epidemiology (STROBE) reporting guideline.

### Data Sources and Measures

Deidentified EMRs included demographic information (age, sex, and ethnicity) and validated MMR vaccination dates. Ethnicity was self-identified by a parent or guardian at the time of registration, selected from a standardized list of 104 categories (eTable in Supplement 1). Children explicitly identified as Somali were classified accordingly; all others were considered non-Somali descent. Somali descent was selected a priori as a subgroup of interest due to the disproportionate concentration of outbreak cases.^6^

### Exposure and Main Outcome Measures

The exposure of interest was active care within the PCN at each time point, which enabled the determination of vaccination status. Vaccination status was assessed using CDC surveillance definitions. MMR1 was considered valid if administered at or after 12 months of age. MMR2 was considered valid if administered at least 28 days after MMR1 and before the age of 84 months (7 years). A 4-day grace period was not applied.

Three outcomes were evaluated independently at each time point, using age-eligible subgroups. Timely MMR1 was calculated as children who received MMR1 between 12 and 16 months divided by children 16 months or older. Timely MMR2 was calculated as children who received MMR2 between 28 days and 84 months divided by children 84 months or older. At least 1 MMR vaccine disease was calculated as children who received 1 or more valid MMR dose between 12 and 84 months divided by children 84 months or older.

### Statistical Analysis

Vaccination coverage for each outcome was calculated as the proportion of eligible children who met defined criteria. Time was modeled as a 3-level categorical variable (0, 12, and 20 months), with outbreak onset (*t* = 0) as the reference. Changes in coverage at 12 and 20 months were compared with baseline using binomial generalized linear models with an identity link and robust SEs. Results were expressed as absolute risk differences with corresponding 95% CIs and 2-sided *P* values, with *P* < .05 considered statistically significant. Subgroup-specific and between-group estimates were reported accordingly.

Age was structurally adjusted for by restricting each analysis to children who were age-eligible for MMR1 or MMR2 at each time point, in accordance with CDC-recommended thresholds. Analyses were stratified by children of Somali descent, selected a priori due to their disproportionate involvement at outbreak onset and longstanding disparities in MMR coverage.^6^ Sex was not included as a covariate because it is not a known factor associated with MMR vaccine uptake in this population. Further geographic stratification was not performed because the study population was already limited to the central Ohio area served by the PCN clinics, the setting of the outbreak response.

Analyses were conducted using R version 4.5.1 (R Foundation for Statistical Computing). Visualizations were generated using Python, version 3.10 (Python Software Foundation).

## Results

### PCN Vaccination Coverage 

At the onset of the outbreak (*t* = 0 months), the cohort included 133 476 children younger than 15 years; this number increased to 143 720 at 12 months (*t* = 12) and to 149 092 at 20 months (*t* = 20) (median [IQR] age, 7.96 [4.23-11.50] years; 76 469 [51.3%] male and 72 618 [48.7%] female; 12 880 [8.6%] Somali descent and 136 212 [91.4%] non-Somali descent) (eTable in Supplement 1). Each time point reflected a reconstructed cross-sectional sample (Table 1).

**Table 1.** MMR Vaccination Coverage at 0, 12, and 20 Months With Risk Differences Compared With Baseline

Time point, mo	Primary care network patients, No./total No. (%)	Risk difference vs baseline (95% CI), percentage points	*P* value^a^
Timely MMR1^b^			
0	66 143/123 490 (53.6)	NA	NA
12	71 588/133 067 (53.8)	0.2 (–0.2 to 0.6)	.22
20	74 157/138 301 (53.6)	0.1 (–0.3 to 0.4)	.76
Timely MMR2^c^			
0	44 210/76 410 (57.9)	NA	NA
12	48 697/81 822 (59.5)	1.6 (1.0 to 2.2)	<.001
20	50 875/84 576 (60.2)	2.3% (1.7 to 2.8)	<.001
At least 1 MMR vaccine dose^d^			
0	59 049/76 410 (77.3)	NA	NA
12	63 710/81 822 (77.9)	0.6 (0.2 to 1.0)	.006
20	65 891/84 576 (77.9)	0.6 (0.2 to 1.0)	.003

Abbreviations: MMR, measles-mumps-rubella; MMR1, first MMR vaccine dose; MMR2, second MMR vaccine dose; NA, not applicable.

^a^ Compared with baseline.

^b^ Children who received MMR1 between 12.0 and younger than 16.0 months among those 16.0 months or older.

^c^ Children who received MMR2 at least 28 days after their first MMR1 and before reaching 84.0 months of age among those 84.0 months or older.

^d^ Children who received at least 1 valid MMR dose between 12.0 and younger than 84.0 months of age among those 84.0 months or older.

Timely receipt of MMR1 remained unchanged: 53.6% (66 143 of 123 490) at 0 months, 53.8% (71 588 of 133 067) at 12 months, and 53.6% (74 157 of 138 301) at 20 months. Compared with baseline, the risk difference at 12 months was 0.2 percentage points (95% CI, –0.2 to 0.6 percentage points; *P* = .22) and at 20 months was 0.1 percentage points (95% CI, –0.3 to 0.4 percentage points; *P* = .76).

Timely receipt of MMR2 increased from 57.9% (44 210 of 76 410) at 0 months to 59.5% (48 697 of 81 822) at 12 months and 60.2% (50 875 of 84 576) at 20 months. These increases were statistically significant, with a risk difference of 1.6 percentage points at 12 months (95% CI, 1.0-2.2 percentage points; *P* < .001) and 2.3 percentage points at 20 months (95% CI, 1.7-2.8 percentage points; *P* < .001).

Coverage with at least 1 valid MMR dose by 84 months of age was 77.3% (59 049 of 76 410) at 0 months, 77.9% (63 710 of 81 822) at 12 months, and 77.9% (65 891 of 84 576) at 20 months. These increases were also statistically significant compared with baseline, with a risk difference of 0.6 percentage points at both 12 months (95% CI, 0.2-1.0 percentages points; *P* = .006) and 20 months (95% CI, 0.2-1.0 percentage points; *P* = .003).

### Vaccination Coverage by Somali Descent 

At 0 months, children of Somali descent represented 11 689 of 123 490 (9.5%) of the analytic cohort; this number decreased to 12 055 of 133 067 (9.1%) at 12 months and 12 156 of 138 301 (8.8%) at 20 months (Table 2; Figure). Timely MMR1 coverage remained significantly lower among children of Somali descent. At 0 months, 35.7% (4171 of 11 689) received a timely first dose compared with 55.4% (61 972 of 111 801) among children of non-Somali descent—a risk difference of –19.7 percentage points (95% CI, –20.7 to –18.8 percentage points; *P* < .001). This gap widened to –20.9 percentage points at 12 months and –22.1 percentage points at 20 months (both *P* < .001).

**Table 2.** MMR Vaccination Coverage by Somali and Non-Somali Descent Groups With Risk Differences

Time point, mo	Patients with vaccination coverage, No./total No. (%)		Risk difference, percentage points (95% CI)	*P* value^a^
	Somali descent	Non-Somali descent		
Timely MMR1^b^				
0	4171/11 689 (35.7)	61 972/111 801 (55.4)	−19.7 (−20.7 to −18.8)	<.001
12	4191/12 055 (34.8)	67 397/121 012 (55.7)	−20.9 (−21.8 to −20.0)	<.001
20	4070/12 156 (33.5)	70 087/126 145 (55.6)	−22.1 (−23.0 to −21.2)	<.001
Timely MMR2^c^				
0	3983/6954 (57.3)	40 227/69 456 (57.9)	−0.6 (−1.9 to 0.6)	.30
12	4321/7350 (58.8)	44 376/74 472 (59.6)	−0.8 (−2.0 to 0.4)	.19
20	4525/7583 (59.7)	46 350/76 993 (60.2)	−0.5 (−1.7 to 0.6)	.37
At least 1 MMR vaccine dose^d^				
0	5357/6954 (77.0)	53 692/69 456 (77.3)	−0.3 (−1.3 to 0.8)	.61
12	5732/7350 (78.0)	57 978/74 472 (77.9)	0.1 (−0.9 to 1.1)	.79
20	5938/7583 (78.3)	59 953/76 993 (77.9)	0.4 (−0.5 to 1.4)	.38

Abbreviations: MMR, measles-mumps-rubella; MMR1, first MMR vaccine dose; MMR2, second MMR vaccine dose.

^a^ Compared with baseline.

^b^ Children who received MMR1 between 12.0 and younger than 16.0 months among those 16.0 months or older.

^c^ Children who received MMR2 at least 28 days after their first MMR1 and before reaching 84.0 months of age among those 84.0 months or older.

^d^ Children who received at least 1 valid MMR dose between 12.0 and younger than 84.0 months of age among those 84.0 months or older.

**Figure.**
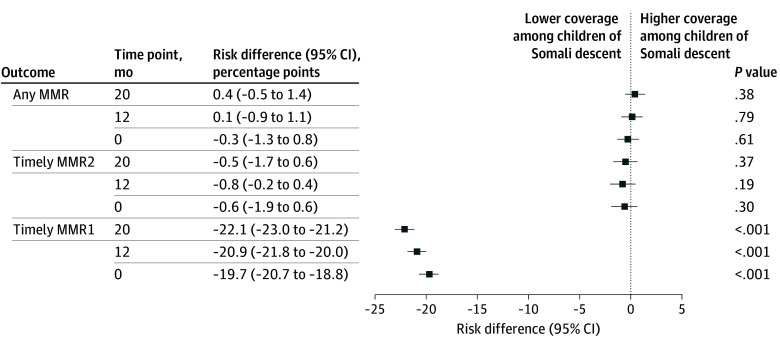
Measles-Mumps-Rubella (MMR) Vaccination Coverage Gaps by Ethnicity and Time Point Risk differences and 95% CIs are shown for 3 MMR vaccination outcomes (timely first MMR vaccine dose [MMR1], timely second MMR vaccine dose [MMR2], and receipt of at least 1 MMR vaccine dose) by children of Somali descent vs children of non-Somali descent at baseline, 12 months, and 20 months. Negative values indicate lower coverage among children of Somali descent compared with children of non-Somali descent.

For timely MMR2, no statistical difference was observed by ethnicity at 20 months: 59.7% (4525 of 7583) among children of Somali descent vs 60.2% (46 350 of 76 993) children of non-Somali descent (risk difference, –0.5 percentage points; 95% CI, –1.7 to 0.6 percentage points; *P* = .37). Receipt of at least 1 MMR dose by 84 months was comparable across groups. At 20 months, 78.3% of children of Somali descent (5938 of 7583) and 77.9% of children of non-Somali descent (59 953 of 76 993) had received at least 1 dose (risk difference, 0.4 percentage points; 95% CI, –0.5 to 1.4 percentage points; *P* = .38).

## Discussion

This repeated cross-sectional study found that MMR vaccination coverage remained well below the herd immunity threshold across all key metrics up to 20 months after a large postelimination measles outbreak in central Ohio. Despite targeted containment efforts during the outbreak, timely MMR1 coverage remained unchanged, and only modest gains were observed in timely MMR2 and overall vaccine receipt. Wide immunity gaps persisted throughout the pediatric population.

Children of Somali descent—who were disproportionately affected during the early phase of the outbreak^6^—consistently exhibited lower timely MMR1 coverage across all time points. These disparities suggest missed opportunities for early vaccination, which structural, cultural, or informational barriers may influence. However, differences narrowed by MMR2 eligibility, likely due to age-based catch-up, prekindergarten visits, or school-entry requirements. This convergence emphasizes the potential equity-promoting impact of age-based public health policies and underscores the importance of earlier engagement to close primary series gaps.

Still, the broader PCN population also showed substantially underwhelming coverage. At the onset of the outbreak, only 53.6% of eligible children received timely MMR1, and even 20 months later, this figure remained unchanged. Timely MMR2 coverage and receipt of at least 1 MMR dose by 84 months of age increased slightly but remained below the 93% critical threshold necessary to sustain measles elimination.^3^ These findings suggest that public health messaging and interventions implemented during the outbreak did not translate into sustained system-level improvements in vaccination uptake.

Jurisdictional estimates reported by CPH during the outbreak (80%-90% coverage) lacked methodological detail and were not stratified by age or dose.^7^ In contrast, this study leveraged individual-level EMR data from a large, continuously engaged pediatric population to provide a more granular and longitudinal assessment. The timely MMR2 coverage of 57.9% observed at outbreak onset aligns with prior modeling by Martoma et al,^3^ which estimated 2-dose coverage at 53% (95% credible interval, 21%-77%). However, the estimates are not directly comparable because the modeled population included children who were not yet age-eligible for MMR vaccination.^3^

The persistence of low coverage metrics spotlights how the central Ohio pediatric population is at risk for future measles outbreaks. With more than 149 000 children in care, the PCN represents a substantial population in which even minor lapses in herd immunity can result in widespread transmission. These risks are not limited to any single community. Although children of Somali descent remain a priority for outreach, the data clearly show that the broader population is also underimmunized and susceptible.

This study also highlights systemic limitations in the US vaccination surveillance infrastructure. However, integration with statewide immunization information systems may be constrained by technical incompatibilities, inconsistent reporting standards, and legal or administrative barriers. Strengthening this infrastructure is essential to enable coordinated, real-time vaccine surveillance. Our findings support the role of EMR-based surveillance as a complement to existing public health tools and underline the need for national investment in real-time, integrated immunization monitoring.

Although measles was declared eliminated in the US a quarter-century ago^4^—defined as the absence of continuous endemic transmission for 12 months or more—recent large postelimination outbreaks in Ohio and Texas signal decreasing population immunity and increasing threats from misinformation, vaccine hesitancy, and reduced public confidence. As of mid-2025, the US has reported 1309 measles cases^8^—the highest annual total in more than 33 years^9^—and 3 confirmed deaths,^8^ the first measles-related deaths in a decade.^10^ The ongoing Texas outbreak, projected to exceed 12 months,^10^ threatens to reverse US measles elimination and endangers elimination status across the World Health Organization Region of the Americas.^11^

Given the continued resurgence of measles in underimmunized US communities, these findings underscore the urgent need to invest in proactive surveillance and targeted outreach. Sustaining elimination will require not only timely outbreak response but also durable, equity-focused improvements in routine vaccination coverage.

### Limitations

This study has limitations. First, the analysis was limited to a single pediatric PCN in central Ohio. Although this may constrain generalizability to other populations or regions, the use of integrated electronic health records from a large, diverse, and regionally representative network strengthens its relevance for similar urban settings and demonstrates a scalable surveillance model.

Second, only children actively engaged in care—defined as having a well-child visit in the preceding 24 months—were included. This may modestly overestimate coverage compared with the general pediatric population because children not engaged in care may have lower vaccination rates.

Third, vaccination data relied on EMRs that undergo reconciliation with state immunization registries and external practitioner documentation. Nonetheless, historical doses may be underreported, especially for children who transferred into the network, potentially resulting in conservative coverage estimates.

Fourth, ethnicity data were derived from structured registration fields based on guardian self-report. Somali descent was captured as a discrete category, but other ethnic subgroups were not analyzed, limiting insights into broader sociodemographic variation. Moreover, Somali status may be misclassified or missing in some cases.

Fifth, this study did not assess behavioral, structural, or informational factors contributing to underimmunization, such as vaccine hesitancy, mistrust, or health care access barriers, that likely shaped coverage patterns during and after the outbreak. Sixth, follow-up was limited to 20 months after onset due to study timing and data availability.

Seventh, as a repeated cross-sectional study, this analysis did not follow up individual children over time and therefore could not assess changes in vaccination status at the individual level or evaluate factors associated with vaccination uptake. As such, it cannot establish causality or directly attribute changes in coverage to the outbreak or public health interventions. Unmeasured secular trends may also have influenced the observed outcomes.

## Conclusions

In this repeated cross-sectional study of an extensive pediatric PCN in central Ohio, MMR vaccination coverage remained well below the herd immunity threshold across all primary metrics 20 months after a major postelimination measles outbreak. Although children of Somali descent exhibited the lowest rates of timely MMR1 receipt, underimmunization was widespread and not limited to a single ethnic subgroup. These findings suggest the urgent need for targeted, data-informed public health strategies and ongoing surveillance to close immunity gaps and sustain measles elimination in the US.

## Notes

Supplement 1. **eTable.** Ethnicity Categories

Supplement 2.
Data Sharing Statement
