# Supplementary material for: Measles Vaccination Coverage After a Postelimination Outbreak
Source: JAMA Netw Open. 2025 Sep 24;8(9):e2533732. doi: 10.1001/jamanetworkopen.2025.33732 (PMC12461437; doi:10.1001/jamanetworkopen.2025.33732)
Supplement: Supplement 1. — eTable. Ethnicity Categories [file jamanetwopen-e2533732-s001.pdf]

# Supplemental Online Content

Martoma RA, Martoma JC, Majumder MS. Measles vaccination coverage after a postelimination outbreak. *JAMA Netw Open*. 2025;8(9):e2533732. doi:10.1001/jamanetworkopen.2025.33732

**eTable.** Ethnicity Categories

This supplemental material has been provided by the authors to give readers additional information about their work.

© 2025 Martoma RA et al. *JAMA Network Open.*

**eTable.** Ethnicity Categories

| Ethnicity Category | | | |
|---|---|---|---|
| Afghanistani/Afghan/Afghani | Colombian | Iraqi | Salvadoran |
| African | Costa Rican | Irish | Scottish |
| Alaskan | Cuban | Israeli | Senegalese/Senegal |
| Albanian | Dominican Islander | Italian | Sierra Leonean |
| American | Dominican | Jamaican | Singaporean |
| Appalachian | Dutch | Japanese | Somali |
| Arab/Arabic | Eastern African | Kenyan | South American |
| Asian Indian | Eastern European | Korean | South American Indian |
| Assyrian | Ecuadorian | Kurdish/Kurd | Spanish |
| Bahamian | Egyptian | Laotian/Lao | Sri Lankan |
| Bangladeshi | Eritrean | Lebanese | Syrian |
| Bantu | Ethiopian | Liberian | Taiwanese |
| Bolivian | European | Malaysian | Thai |
| Brazilian | Filipino | Mexican | Trinidadian |
| British/English | French | Mexican American Indian | Turkish/Turk |
| Bulgarian | Gambian | Middle Eastern/North African | Ugandan |
| Burmese | German | Mnong | Ukrainian |
| Cambodian | Ghanaian/Ghanian | Moroccan | Uzbekg/Uzbek |
| Cameroonian | Greek | Nepalese/Nepali | Vietnamese |
| Canadian | Guatemalan | Nigerian | West Indian |
| Cape Verdean | Guinea | Pakistani | West African |
| Caribbean Islander | Haitian | Palestinian | Zairean |
| Central African | Honduran | Polish/Pole | Guardian Unavailable to Ask |
| Central African Republic | Hong Kong | Portuguese | Multi Ethnicity |
| Central American | Indonesian | Puerto Rican | Patient/Family Declined |
| Chinese | Iranian | Russian | Other Ethnicity |

This table lists the 104 ethnicity categories that were available for guardians to select during the data collection process.

© 2025 Martoma RA et al. *JAMA Network Open.*
