# Supplementary material for: Measles Vaccination Coverage After a Postelimination Outbreak
Source: JAMA Netw Open. 2025 Sep 24;8(9):e2533732. doi: 10.1001/jamanetworkopen.2025.33732 (PMC12461437; doi:10.1001/jamanetworkopen.2025.33732)
Supplement: Supplement 2. — Data Sharing Statement [file jamanetwopen-e2533732-s002.pdf]

Data Sharing Statement

Martoma. Measles Vaccination Coverage After a Postelimination Outbreak. *JAMA Netw Open*. Published September 24, 2025. doi:10.1001/jamanetworkopen.2025.33732

Data
Data available: No

Additional Information
Explanation for why data not available: In accordance with institutional privacy policies, only deidentified, aggregated summary tables have been provided. Individual-level patient data will not be shared.
